# Spontaneous bilateral tubal ectopic pregnancy preoperatively diagnosed by the ultrasound: a case report

**DOI:** 10.1186/s12884-023-05458-z

**Published:** 2023-02-23

**Authors:** Elham Eghbali, Mojtaba Azari, Ali Jafarizadeh, Samin Alihosseini

**Affiliations:** 1 Medical Radiation Sciences Research Group, Tabriz University of Medical Sciences, Tabriz, Iran; 2 Student Research Committee, Tabriz University of Medical Sciences, Tabriz, Iran

**Keywords:** Ectopic pregnancy, Bilateral adnexal mass, Assisted reproduction, Ultrasound scan, Case report

## Abstract

**Background:**

Bilateral ectopic pregnancy is extremely rare, with a tremendous maternal mortality and morbidity risk, requiring rapid diagnosis and management. This condition is usually diagnosed during surgery, as radiologists may not pay enough attention to the contralateral side of interest. Therefore, reminding of this rare but emergent situation can be beneficial for both radiologists and gynecologists. Here we report a case of bilateral ectopic pregnancy, which was first diagnosed with ultrasound and was confirmed during laparoscopy.

**Case presentation:**

A 34 years old woman complaining of light vaginal bleeding at 6 weeks of gestation by her last menstrual period presented to our institute. The serum *β*-HCG levels were analyzed and followed during patient’s admission. Unfortunately, serum levels weren’t decreasing and blood test titration before surgery were as: 851,894,975 IU/l (checked daily and not every 48 h because of patient’s status and being bilateral). There was no evidence of intrauterine pregnancy at the transvaginal ultrasound, but heterogeneous adnexal masses were seen at both adnexa, suspected of bilateral ectopic pregnancy. She underwent laparoscopic exploration, which confirmed the diagnosis. Bilateral salpingostomy was done to preserve fertility, and the patient’s recovery was uneventful.

**Conclusions:**

Even with a unilateral report of ectopic pregnancy preoperatively in ultrasonography, surgeons should always be aware of the probability of bilateral ectopic pregnancies anytime facing susceptible cases, especially in patients with known risk factors. Also, it is an important reminder for radiologists to check both adnexa when facing a unilateral adnexal mass resembling ectopic pregnancy.

## Background

When a fertilized ovum implants outside of the uterine cavity, ectopic pregnancy (EP) happens. The estimated incidence of EP is 1 to 2% [1], and 2.7% of pregnancy-related deaths are attributed to ruptured EP [2]. The fallopian tubes are where EPs most frequently occur, accounting for about 97%. Bilateral tubal ectopic pregnancy (BTP) is a very rare type of EP, with a reported incidence of 1/200,000 uterine pregnancies and 1/725–1/1580 EPs [3]. Due to the increasing use of assisted reproduction techniques (ART), use of intrauterine contraceptive devices (IUD), pelvic inflammatory disease (PID), and a history of previous EP or tubal surgery, a threefold increase in the incidence of BTP has been reported in the recent decades [4]. BTP is clinically indistinguishable from a unilateral tubal ectopic pregnancy, and the majority of instances are detected accidentally at the surgery. Considering the significant risk of morbidity and mortality, the diagnosis and appropriate management of a BTP are critical [5].

We report a case of spontaneous bilateral tubal EP that was diagnosed preoperatively with ultrasonography and managed successfully at our institute.

## Case presentation

**Fig. 1:**
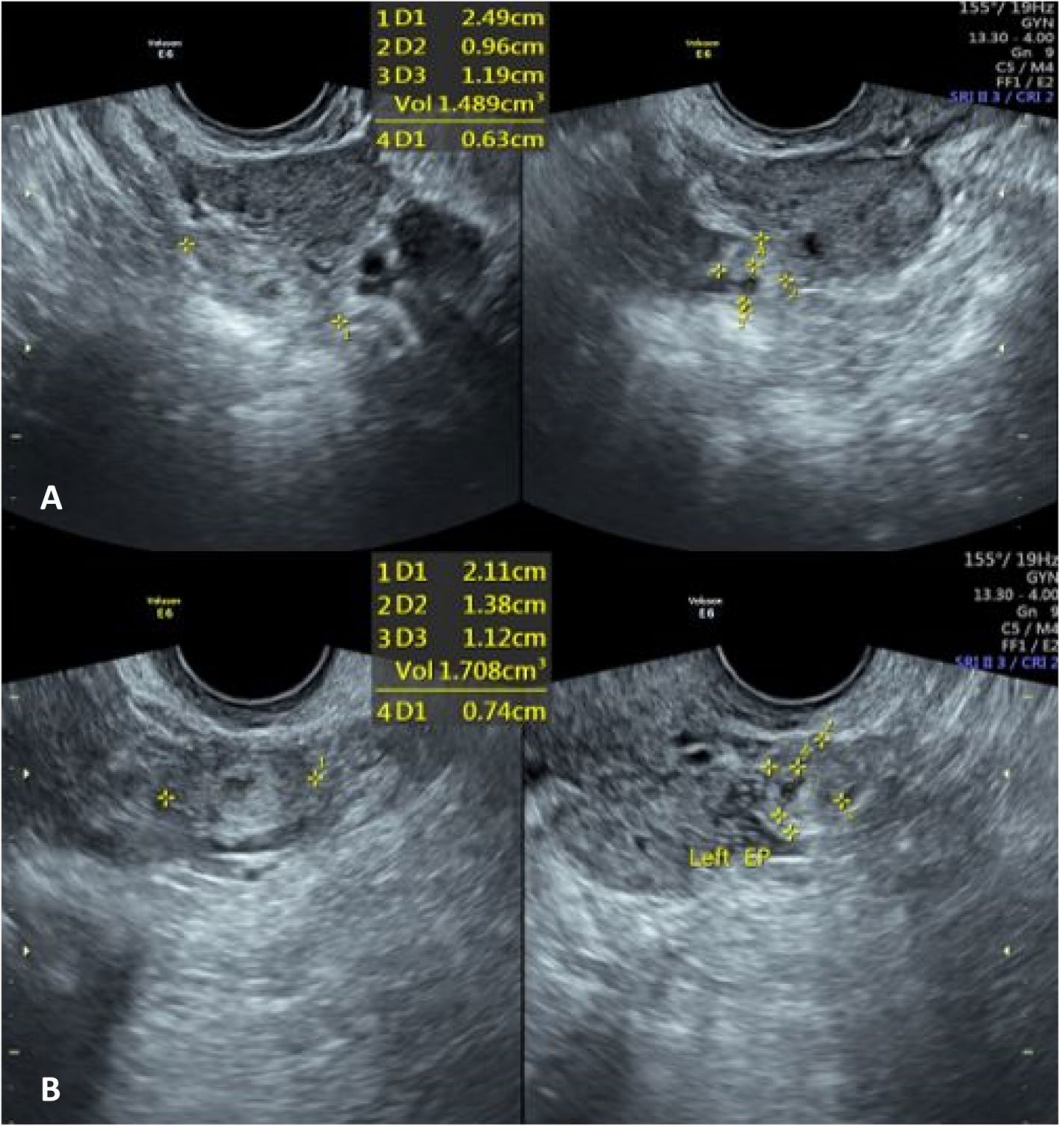
A heterogeneous tubular mass measuring 24*9*11 ^mm^ and 1.4 ^cc^ is seen adjacent to the right ovary (**A**). Another similar mass measuring 21*13*11^mm^ and 1.7 ^cc^ is seen near the left ovary (**B**), suspected of bilateral unruptured ectopic pregnancies

A 34-year-old gravida 2 para 1 woman presented with light vaginal bleeding at 6 weeks of gestation by the last menstrual period. Her medical history revealed hypothyroidism and secondary infertility for 9 years, with a cesarean section in her past gynecological and surgical history. She had no history of sexually transmitted infections (STI) or any ARTs utilization. The patient was hemodynamically stable. Laboratory investigations showed a serum beta-human chorionic gonadotropin (*β*-HCG) titration of 851,894 and 975 IU/l, (checked daily and not every 48 hours because of patient’s status and being bilateral). Transvaginal ultrasound (Philips affinity 70 sonography device and a 2 to 5 MHz probe) found no evidence of intrauterine gestation, no fluid in the cul-de-sac, and approximately 13 mm adnexal mass in the right and 21 mm in the left (Fig. 1).

**Fig. 2:**
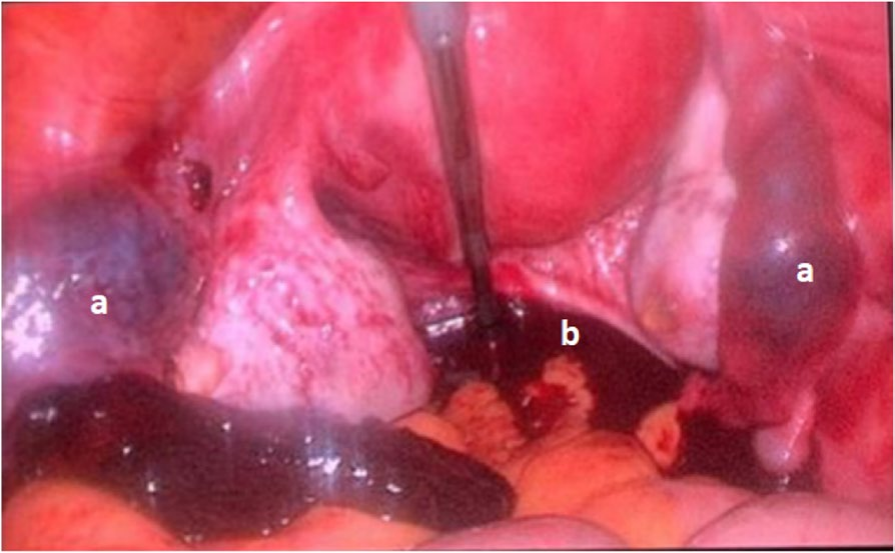
Bilateral tubal ectopic pregnancies (**a**) and a small amount of blood (**b**) in the cul-de-sac are seen

Due to the possibility of bilateral tubal pregnancy, laparoscopic exploration was performed after the patient and her husband informed consent. The right fallopian tube was found to contain a mass measuring 20 mm in the isthmic part. The left fallopian tube also showed a 21 mm mass in the ampullary region, also suggestive of an ectopic tubal pregnancy. Both masses were unruptured. A small amount of blood was also discovered in the cul-de-sac (Fig. 2).

**Fig. 3:**
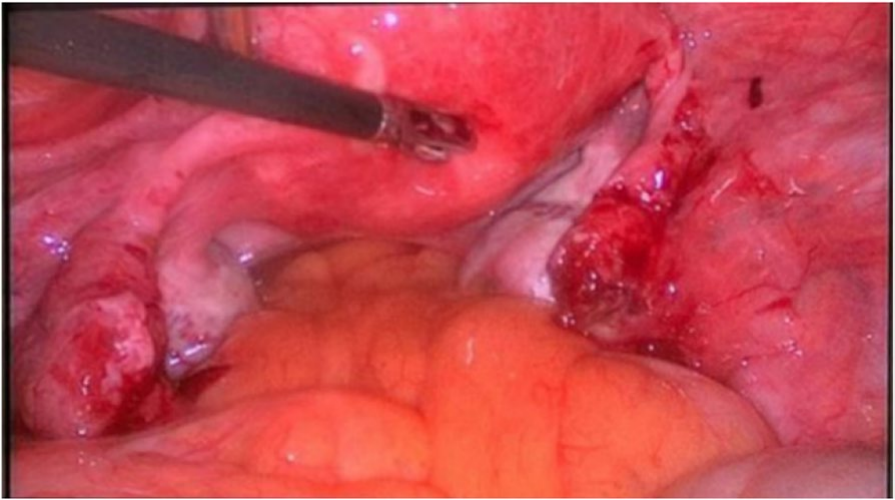
Laparoscopic image after salpingotomy shows the removal of bilateral tubal masses

The patient underwent bilateral salpingostomy to preserve fertility, as the fallopian tubes were intact with unruptured pregnancies. The pathological examination confirmed the presence of chorionic villi in the extracted contents of both tubes, and her recovery period was smooth. The patient was followed up closely after discharge. Four weeks postoperatively, serum *β*-HCG levels had a decreasing pattern with a dynamics of 172,125,67,23. On the fifth and sixth week after surgery *β*-HCG levels were 1.5 and 1.2 (Fig. 3).

## Discussion

EP is one of the life-threatening emergencies of gynecology and obstetrics, with an incidence of 1% and rising prevalence [6]. Bilateral tubal pregnancy is extremely rare and simultaneous bilateral tubal pregnancy (SBTP) is the rarest form in the absence of ovulation induction procedures and ART, with an incidence of one per 200,000 live births [7]. A substantial increase in BTP occurrence has been observed in recent decades, probably due to the growing utilization of ARTs and ovulation induction. Some other risk factors for extrauterine pregnancies include a history of sexually transmitted infections, multiple sexual partners, intrauterine devices, smoking, hormonal contraception, pelvic surgery, previous ectopic pregnancy, history of infertility, damage of the fallopian tubes, and in utero exposure to diethylstilbestrol [8, 9]. Our presented case had no ARTs application history or other relevant risk factors. Several theories have described the mechanism of BTP occurrence. Simultaneous multiple ovulation, sequential impregnation, and transperitoneal migration of trophoblastic cells from one extrauterine pregnancy to the other tube with implantation there are the suggested theories [9].

The most common presentation of an EP is the classic triad of amenorrhea, vaginal bleeding, and abdominal pain. Early diagnosis of tubal EP is essential to prevent complications like rupture, intraabdominal bleeding, and subsequently maternal mortality and morbidity [10]. Most EPs occur in the fallopian tubes, with the ampulla being the most common site. Bilateral versus unilateral EPs are usually not clinically discernible [11]. Serum *β*-HCG levels measurement is not reliable for the diagnosis of a BTP, and as an extrauterine pregnancy is often diagnosed in the absence of intrauterine pregnancy rather than direct visualization of the ectopic itself in the ultrasound examination, a BTP is mostly diagnosed intra-operatively. A review of the literature revealed that only very few cases of BTP were detected preoperatively by ultrasound [12–14]. In these situations, the experience and skill of the sonographer is important for early diagnosis before diagnostic surgery. Sonographers should be aware of this fault that diagnosing in one side of adnexa shouldn’t satisfy the sonographer from additional evaluation and searching the contralateral adnexum [15]. Thankful for the proficiency and conversance of our radiologist, we were able to suspect a bilateral tubal pregnancy preoperatively.

Good management of a BTP relies on early presentation, a high index of suspicion, detailed ultrasound scan, precise intraoperative examination of the contralateral tube, histological confirmation, and appropriate patient counseling, regardless of whether it is spontaneous or induced [16]. Depending on the patient’s clinical state, the site of EP, whether it is ruptured or not, the patient’s willingness to preserve fertility, and available facilities, EP cases may be managed by medical, surgical, or expectant methods [17]. Medical management by methotrexate administration may be applied for suspected unilateral cases meeting the eligibility criteria, closely monitoring with serial *β*-HCG until the complete resolution of pregnancy. Since ultrasound examination has limitations for the definite diagnosis of a BTP and there are no studies explaining dosing regimen and efficacy of methotrexate administration for BTPs, medical management seems not suitable for a suspected BTP [11]. Reviewing the literature shows only one case of a spontaneous BTP diagnosed preoperatively by ultrasound examination and successfully treated by two vaginal ultrasound-guided intratubal methotrexate injections [13]. Other rare reported cases could see the BTP after a period of time spent and patient being unwell and needing urgent laparotomy because of hemodynamic instability [18]. Ruptured ectopic pregnancy suspicion, hemodynamically unstable patients, and medical management contraindications require surgical interventions [11]. Surgical management may be through radical salpingectomy or conservative linear salpingotomy [19]. Since a BTP is almost always diagnosed intraoperatively, detailed and thorough exploration of the whole pelvic cavity is essential to prevent a missed diagnosis and the risk of repeated surgery. Although the fertility rate decreases in patients with an EP history, salpingotomy is commonly performed in the case of unruptured EPs, as our patient underwent bilateral salpingostomy [20]. However, a multicenter, randomized controlled trial conducted by Mol et al. demonstrated that compared to salpingectomy, salpingotomy does not considerably increase fertility prospects [21]. In the case of salpingostomy and tubal conservation, careful follow-ups with serial measurement of *β*-HCG are necessary to rule out the risk of persistent trophoblast until complete resolution [22]. In 6–16% of women who have had previous ectopic pregnancies, recurrent ectopic pregnancies occur [23], and many women fail to conceive even after successful reconstructive tubal surgery for unknown reasons [16, 24]. For patients undergoing bilateral salpingectomy, assisted reproductive technologies are the only option for future fertility [25]. The limitation of our study can be related to the model of the ultrasound device, and it seems that the quality and model of the ultrasound device have a significant impact on the radiologist’s diagnosis and, finally the management of the patient. So, studies based on the model of the ultrasound devices may be helpful and necessary.

## Conclusion

Bilateral tubal ectopic pregnancy is exceptionally uncommon and is associated with high mortality and maternal complications. Therefore, timely diagnosis and appropriate management are vital. In the present case, early presentation of the patient, high suspicion of a BTP preoperatively in the ultrasound scan, and detailed inspection of the contralateral tube resulted in the successful management of this life-threatening condition.

## Data Availability

All available data are presented in this article and if more explanations are needed, contact the corresponding author.

## Abbreviations

EP: Ectopic pregnancy
BTP: Bilateral tubal ectopic pregnancy
ART: Assisted reproduction techniques
IUD: Intrauterine contraceptive devices
PID: Pelvic inflammatory disease
STI: Sexually transmitted infections
SBTP: Simultaneous bilateral tubal pregnancy
*β*-HCG: Beta-human chorionic gonadotropin
